# Establishment of a New-Generation National Reference Material System for Fragile X Syndrome Using Targeted Long-Read Sequencing

**DOI:** 10.3390/genes17060656

**Published:** 2026-06-02

**Authors:** Mi Zhang, Wenxin Zhang, Fei Gao, Huiying Fang, Li Zhang, Yaning Qi, Wei Zhang, Peiwen Xu, Jie Li, Shoufang Qu

**Affiliations:** 1 National Institutes for Food and Drug Control, Beijing 100050, China; zhangmi@nifdc.org.cn (M.Z.); zhangwenxin@nifdc.org.cn (W.Z.); feigao@nifdc.org.cn (F.G.); qiyaning@nifdc.org.cn (Y.Q.); zhangwei88@nifdc.org.cn (W.Z.); 2 State Key Laboratory of Drug Regulatory Science, Beijing 100050, China; 3 Berry Genomics Corporation, Beijing 102200, China; fanghuiying225@berrygenomics.com (H.F.); zhangli@berrygenomics.com (L.Z.); 4 State Key Laboratory of Reproductive Medicine and Offspring Health, Center for Reproductive Medicine, Shandong University, Jinan 250000, China; xupeiwen@sduivf.com (P.X.); lijie@sduivf.com (J.L.); 5 National Research Center for Assisted Reproductive Technology and Reproductive Genetics, Shandong University, Jinan 250000, China

**Keywords:** fragile X syndrome, targeted long-read sequencing, reference materials, CGG repeats, AGG interruptions

## Abstract

Background: Fragile X syndrome (FXS) is the most common monogenic cause of inherited intellectual disability and is primarily caused by CGG repeat expansion in the *FMR1* gene. Conventional diagnostic methods have limited precision for sizing long repeat sequences and cannot resolve AGG interruptions, which are critical for comprehensive risk assessment. Existing national FXS reference materials are based on conventional methods and provide limited molecular information. Methods: We developed a targeted long-read sequencing assay for comprehensive *FMR1* characterization, termed tLRS-*FMR1*, and applied it to a panel of 22 national FXS reference materials. Results: The tLRS-*FMR1* assay demonstrated 100% concordance with standard methods while overcoming key limitations of conventional approaches. It enabled precise quantification of CGG repeat numbers, including full mutations (>200 repeats) that were only qualitatively reported by traditional techniques and provided comprehensive mapping of AGG interruption patterns. The assay showed high reproducibility, with 100% genotyping concordance across intra- and inter-assay replicates and achieved a detection limit of 3 ng/μL. Conclusions: This study successfully developed tLRS-*FMR1* and established a new-generation national FXS reference material system with expanded molecular information and improved precision, providing a foundation for advancing the standardization and accuracy of FXS molecular diagnosis.

## 1. Introduction

Fragile X syndrome (FXS) is the most common monogenic cause of inherited intellectual disability and autism spectrum disorder (ASD) [1]. In over 99% of cases, it is caused by CGG repeat expansion and abnormal methylation in the fragile X messenger ribonucleoprotein 1 gene (*FMR1*; OMIM:309550) located on the X chromosome, leading to deficiency of the encoded protein FMRP. The remaining cases result from point mutations or deletion mutations in the *FMR1* gene. Based on the CGG repeat number, *FMR1* alleles are classified into four categories: normal (5~44 repeats), intermediate or gray zone (45~54 repeats), premutation (55~200 repeats), and full mutation (>200 repeats) [2,3,4]. Over 99% of males carrying a full mutation develop FXS, manifesting varying degrees of intellectual disability. In contrast, females with a full mutation exhibit high clinical heterogeneity, with approximately 50% developing FXS, typically presenting with milder symptoms than their male counterparts [2,5]. Among premutation carriers, about 40% of males and 8% of females may develop fragile X-associated tremor/ataxia syndrome (FXTAS) [6] after the age of 50, while approximately 20% of female carriers may experience fragile X-associated primary ovarian insufficiency (FXPOI) [7], characterized by symptoms such as amenorrhea, before the age of 40. These premutation-associated manifestations are clinically distinct from the typical neurodevelopmental phenotype observed in full mutation FXS and usually show later onset and/or different clinical features.

Offspring of individuals carrying intermediate alleles may only show minor fluctuations (slight increases or decreases) in the CGG repeat number, with a minimal risk of expansion to a full mutation. Conversely, offspring of female premutation carriers carry a significantly elevated risk of expansion to a full mutation [8]. This risk demonstrates a positive correlation with the maternal CGG repeat count; for instance, the risk is approximately 3% for repeats numbering 55~59, escalating to as high as 100% for repeats numbering 100~200 [9]. Notably, AGG trinucleotide interruptions within the CGG repeats stabilize the sequence and reduce the risk of expansion during transmission, with this effect being most prominent in samples with <70 CGG repeats [9,10,11]. Thus, AGG interruptions are clinically relevant primarily as modifiers of repeat instability and transmission risk, rather than as direct determinants of disease phenotype. The analysis of AGG interruption patterns holds indispensable value for assessing parental transmission risk, predicting offspring prognosis, and guiding reproductive decision-making [12].

However, conventional FXS molecular diagnostic techniques possess significant limitations. Triplet-primed PCR (TP-PCR), while operationally convenient, fails to accurately size long repeat sequences [13], struggles to resolve AGG interruption patterns (particularly in female heterozygotes or mosaic samples) [14], and is susceptible to false-negative results due to interference from flanking region microdeletions [15,16]. Although Southern blot analysis can detect full mutations and methylation status, it is hampered by a cumbersome workflow, low throughput, limited resolution, and an inability to provide AGG interruption information [17,18]. Short-read genome sequencing is also constrained in *FMR1* CGG repeat analysis, because long, GC-rich, and highly repetitive expansions cannot be reliably spanned or directly resolved [19].These technological bottlenecks pose substantial challenges to precise genetic counseling and risk assessment for FXS [12,18]. In recent years, long-read sequencing (LRS) has been applied to FXS testing and has demonstrated significant clinical potential [13,20,21]. Although long-read genome sequencing can resolve *FMR1* repeat expansions and interruption patterns, it requires substantially higher sequencing output and cost than targeted LRS to achieve adequate locus-specific coverage [22]. Targeted LRS, such as the comprehensive analysis of fragile X syndrome (CAFXS), provide a more focused strategy by simultaneously detecting CGG repeat number, AGG interruption patterns, rare intragenic variants, and mosaic proportions in the *FMR1* gene [23], with demonstrated clinical utility in 238 high-risk clinical samples [24].

A critical bottleneck currently impeding the standardized application of this advanced LRS technology is the lag in the genomic DNA reference material system. The existing national reference materials for FXS are primarily characterized using traditional methods (e.g., PCR and Southern blot) [25], and the information they provide is limited. They cannot offer accurate calibration standards for the comprehensive analytical performance of new technologies like CAFXS, particularly for AGG interruption resolution capabilities. To directly address this gap and provide the necessary calibration standards, we developed a targeted long-read sequencing assay for comprehensive *FMR1* characterization (tLRS-*FMR1*). This newly established method was then applied to conduct comprehensive and precise characterization of national FXS reference materials. The goal is to establish a new-generation national reference material system that fulfills the validation requirements of new technologies, thereby laying a solid foundation for enhancing the quality of clinical diagnosis and genetic counseling for FXS.

## 2. Materials and Methods

### 2.1. Study Subjects

A total of 31 candidate samples were evaluated for the development of the FXS reference material system, as detailed in Supplementary Table S1. Among them, 22 samples were selected as reference materials, consisting of 14 positives (P1–P14) and 8 negatives (N1–N8). The positive samples P1, P2, P4, P13, P14, and P6–P10 were acquired from the Coriell Cell Repositories, P3 and P5 were obtained from the Center for Reproductive Medicine at Shandong University, and P11 and P12 were artificial mosaic samples prepared by diluting Coriell DNA of the same gender to achieve different mass fractions. The panel of positive samples encompasses intermediate, premutation, and full mutation genotypes, as well as mosaics involving two or more genotypes. The negative samples N1–N6, representing normal genotypes, were procured from the Coriell Cell Repositories, whereas N7 and N8, comprising non-human Escherichia coli DNA, were purchased from the American Type Culture Collection (ATCC Manassas, VA, USA). Genomic DNA of all samples was extracted using the GenMagBio Kit (BioBasic Inc., Markham, ON, Canada), and its integrity was verified by agarose gel electrophoresis.

### 2.2. PCR Assay for FMR1 Genotyping

The CGG repeat region within the *FMR1* gene was amplified and analyzed using the commercially available AmplideX^®^ PCR/CE *FMR1* Reagent kit (Asuragen, Austin, TX, USA) in accordance with the manufacturer’s protocol. Briefly, TP-PCR was performed, followed by capillary electrophoresis on an ABI 3130 Genetic Analyzer (Thermo Fisher Scientific, Waltham, MA, USA). For data analysis, electropherogram peaks were identified using a threshold of 50 relative fluorescence units as specified in the manufacturer’s guidelines. The precise CGG repeat number was subsequently determined by applying a linear fit algorithm implemented in the GeneMapper software (v4.0, Thermo Fisher Scientific, Waltham, MA, USA).

### 2.3. Targeted LRS Assay for Comprehensive FMR1 Characterization (tLRS-FMR1)

A novel assay (tLRS-*FMR1*) was established in this study to enable the comprehensive detection of CGG repeats and AGG interruptions. The core of this assay is the strategic combination of a robust long-range PCR (LR-PCR) amplification step with subsequent LRS. A pair of primers flanking exon 1 of the *FMR1* gene was designed (Forward: 5′-AATCATTCTAATCAATGTGTCCCC-3′; Reverse: 5′-GAAGCATGTGCATTCCTGAATTT-3′). LR-PCR was performed on all reference materials using a high-fidelity DNA polymerase (KOD FX Neo, TOYOBO, Osaka, Japan). The PCR mixture was supplemented with 5% DMSO and 0.25 M betaine to enhance the amplification efficiency through the GC-rich region. The thermal cycling conditions comprised an initial denaturation at 94 °C for 5 min (1 cycle); followed by 28 cycles of denaturation at 98 °C for 10 s, annealing at 62 °C for 20 s, and extension at 68 °C for 5 min; with a final extension at 68 °C for 10 min (1 cycle). Following amplification, PCR products were ligated to unique barcoded adapters through a one-step end-repair and ligation procedure. Unligated products were removed via exonuclease digestion. The barcoded pre-libraries were purified, quantified using the Qubit dsDNA HS Assay Kit (ThermoFisher Scientific, Waltham, MA, USA), and pooled in equimolar ratios. After additional purification with AMPure PB beads (Pacific Biosciences, Menlo Park, CA, USA), the pooled library was converted to a single-molecule real-time dumbbell (SMRTbell) structure using the Sequel Binding Kit 2.0 and Internal Control Kit 1.0 (Pacific Biosciences). The final SMRTbell library was sequenced on the Sequel II CNDx platform (Berry Genomics Corporation, Beijing, China) under circular consensus sequencing (CCS) mode with a 30 h movie time to generate highly accurate long reads.

The raw sequencing subreads were processed to obtain high-quality CCS reads, debarcoded to individual samples, and aligned to the hg38 reference genome using SMRT Link (v10.1.0.119588, Pacific Biosciences). Reads containing the CGG repeat region plus 100 bp of flanking sequence on each side were extracted via BLAST+ (v2.11.0, NCBI, Bethesda, MD, USA). Flanking sequences were trimmed to retain only the pure CGG repeat tract, and the reads were sorted by length. Quality control thresholds were applied based on the genotype: a minimum of 100 CCS reads for full mutation alleles, 200 for premutation alleles, and 500 for intermediate and wild-type alleles. Repeat regions were annotated with color codes to distinguish CGG and AGG motifs and visualized as waterfall plots. The total CGG repeat number (including AGG interruptions) per read was determined from its length, and allele-specific repeat lengths were identified by applying kernel density estimation to the distribution of repeats.

### 2.4. Evaluation Metrics and Analytical Methods

The performance of the LRS-based national reference panel for FXS was assessed using the following metrics. (1) Positive and negative concordance rates were calculated by comparing the test results of the reference samples (positive and negative) with their known mutation status, as verified by TP-PCR. Discordance is defined according to the previous paper [24]: (i) ≥2 CGG repeats difference for alleles with 5~54 repeats; (ii) ≥5 CGG repeats difference for alleles with 55~199 repeats; or (iii) detection of microdeletions or pathogenic SNVs/indels by the tLRS-*FMR1* that were not identified by the standard method. (2) The robustness of the assay was evaluated by testing all samples in three replicate runs within and across batches and was confirmed by the observed consistency in CGG repeat numbers across all replicates. Inconsistency was defined as (i) ≥2 CGG repeats difference for alleles with 5~54 repeats and (ii) ≥5 CGG repeats difference for alleles with 55~199 repeats. (3) The limit of detection (LOD) was established by testing a series of serial dilutions (2-fold) of a premutation sample. Each dilution level was tested with three replicates. The LOD was defined as the lowest concentration at which a positive result was observed in all replicates.

## 3. Results

### 3.1. Systematic Analysis and Comparative Validation of Reference Materials

A total of 31 candidate samples were evaluated for the development of the FXS reference material system. Based on genotypic diversity, representativeness, and practical panel size, 22 samples were selected as the final reference materials, consisting of 14 positive samples (P1–P14) and eight negative samples (N1–N8), through parallel testing using TP-PCR and the tLRS-*FMR1*. TP-PCR was performed using the AmplideX® PCR/CE FMR1 Kit (Asuragen, Inc., Austin, TX, USA), and tLRS-FMR1 was performed on the Sequel II CNDx platform (Pacific Biosciences, Menlo Park, CA, USA). All data generated by the tLRS-*FMR1* assay passed the quality control criteria. The comparative results are summarized in Table 1, and the *FMR1* genotyping results for the nine samples analyzed but not included in the final panel are summarized in Supplementary Table S2.

In positive samples, initial TP-PCR analysis demonstrated complete concordance between CGG repeat numbers and established data from the Coriell database or known genotypes, with the exception of a single sample (P4). The Coriell database notes inter-laboratory discrepancies for this sample; therefore, the TP-PCR result was used as the reference value in subsequent analyses. The tLRS-*FMR1* validated all genotypes with 100% positive concordance against these reference values. Notably, the assay enabled the precise quantification of CGG repeats in all full mutation-containing samples, overcoming the limitation of TP-PCR, which only provides a qualitative “>200 repeats” designation. For instance, in male sample P8, TP-PCR reported >200 repeats, whereas the tLRS-*FMR1* precisely determined the count to be 311. In the four mosaic samples, tLRS-*FMR1* resolved multiple alleles with different repeat sizes, including 98/311 and 29/505 repeat units in the artificial mosaic samples P11 and P12, respectively, and 23/134/209 and 336/664 repeat units in the authentic mosaic samples P13 and P14, respectively. In negative samples, no abnormal expansions were detected by either method. The tLRS-*FMR1* results were fully consistent with the control results, yielding a negative concordance rate of 100%, which confirms the high specificity of the method.

Furthermore, the tLRS-*FMR1* uniquely resolved the number and positions of AGG interruptions in all samples. The high analytical precision of the assay was demonstrated in female heterozygous samples (e.g., P3 and P7), enabling clear differentiation of the AGG patterns on both X chromosomes—a challenge for TP-PCR due to its limited resolution.

### 3.2. Precise Quantification of CGG Repeats by the tLRS-FMR1

**Figure 1:**
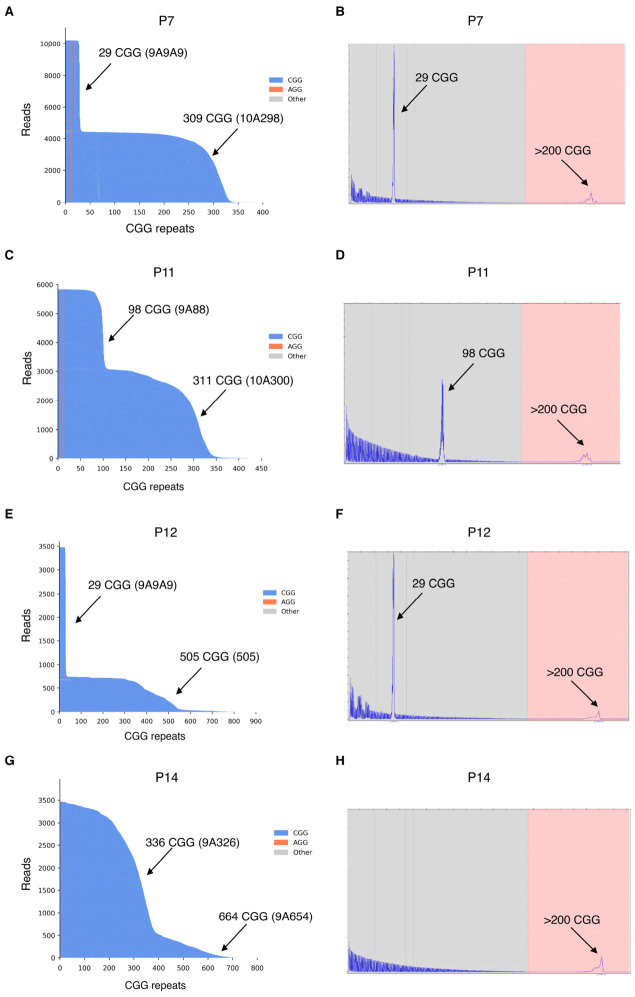
Precise genotyping of CGG repeats by the tLRS-*FMR1* in representative samples carrying full mutation alleles. (**A**) Sample P7 with CGG repeat 29/309 by tLRS-*FMR1*. (**B**) Sample P7 with CGG repeats 29/>200 by PCR assay. (**C**,**D**) Sample P11 with mosaic premutation and full mutation alleles by tLRS-*FMR1* and PCR. (**E**,**F**) Sample P12 with mosaic normal and full mutation alleles by tLRS-*FMR1* and PCR. (**G**,**H**) Sample P14 with mosaic full mutation alleles by tLRS-*FMR1* and PCR. Nucleotides: blue (CGG), orange (AGG), gray (other), The pink shaded region indicates the full mutation range, Arrows highlight the alleles with different CGG repeats.

The tLRS-*FMR1* successfully achieved precise quantification of CGG repeat numbers in all national reference materials, with particular success in accurately determining the repeat counts in eight full mutation-containing samples (P7–P14). Representative results for P7, P11, P12, and P14 are presented in Figure 1, while data for the remaining samples are provided in Supplementary Figure S1. In the female heterozygous sample P7, the tLRS-*FMR1* assay accurately identified a repeat pattern of 29/309 (Figure 1A), whereas TP-PCR only reported 29 and >200 repeats. For the male homozygous full mutation sample P8, the assay precisely determined the repeat number to be 311 (Supplementary Figure S1). Furthermore, the tLRS-*FMR1* resolved repeat patterns of 98/311 and 29/505 in two mosaic samples (P11 and P12, shown in Figure 1C,E, respectively). In the authentic repeat-size mosaic samples P13 and P14, tLRS-*FMR1* identified 23/134/209 (Supplementary Figure S1) and 336/664 repeat units (Figure 1G), respectively. In contrast, TP-PCR could only classify the full mutation alleles in these samples as >200 repeats, without providing precise repeat counts (Figure 1B,D,F,H). It is noteworthy that the full mutation allele in sample P9 was previously estimated by Southern blot analysis to contain 931–940 repeats, while the tLRS-*FMR1* reported a count of 900 repeats. Southern blot provides an estimation derived from molecular weight inference, which may lead to overestimation due to the nonlinear migration of large DNA fragments in gel electrophoresis [26]. In contrast, the tLRS-*FMR1* relies on direct sequence counting and yields higher accuracy. It should also be noted that higher CGG repeat numbers are more prone to generating amplification background, which may lead to minor variations between the detected and actual repeat numbers. For instance, sample P10 showed 508 CGG repeats, while the full mutation allele in sample P12—an artificial mixture of P10 and N2—was determined to have 505 repeats.

### 3.3. The tLRS-FMR1 Reveals Previously Uncharacterized AGG Interruption Patterns

The tLRS-*FMR1* successfully resolved the AGG interruption patterns within the CGG repeat regions across all national reference samples, including complex heterozygous and mosaic alleles (Table 2). The read count, representing the number of reads supporting each specific AGG interruption pattern, exhibited a median of 2444 and a range of 49–30,815, indicating high analytical reliability. These patterns were clearly visualized using high-resolution waterfall plots, which enabled clear observation of the distribution characteristics of AGG interruptions and allele-specific patterns across different samples.

**Figure 2:**
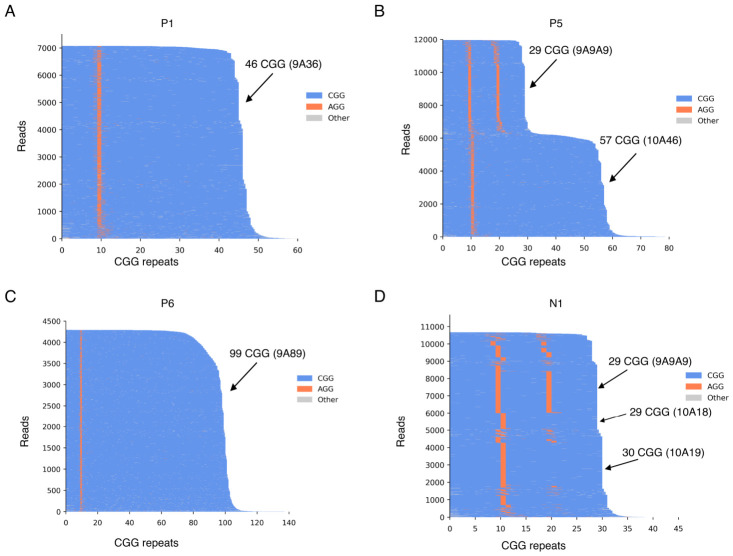
High-resolution mapping of representative AGG interruption patterns. (**A**) Intermediate allele (46 CGG) with one AGG (9A36). (**B**) Heterozygous sample with normal (9A9A9) and premutation (10A46) alleles. (**C**) Premutation allele (99 CGG) with 9A89 pattern. (**D**) Normal sample showing 9A9A9, 10A18, and 10A19 patterns. Nucleotides: blue (CGG), orange (AGG), gray (other), Arrows highlight the alleles with different CGG repeats.

As shown in Figure 2A, sample P1 exhibited an interruption pattern of (CGG)_9_AGG(CGG)_36_ (abbreviated as the 9A36), representing an intermediate allele. Another intermediate sample, P2, contained no AGG interruptions (Supplementary Figure S1). Figure 2B displays the pattern of sample P5, in which the normal allele demonstrated the typical 9A9A9 pattern, a common configuration in normal alleles [24]. The premutation allele in this sample showed a 10A46 pattern; the presence of this AGG interruption may enhance the sequence stability and reduce the risk of expansion to a full mutation. Similar premutation patterns were also observed in samples P3, P4 (Supplementary Figure S1), P6 (Figure 2C), and P11 (Figure 1C). Sample N1 exhibited three distinct interruption patterns: one allele contained both 9A9A9 and 10A18, while the other allele showed a 10A19 pattern (Figure 2D). Notably, AGG interruptions were still retained within the full mutation alleles of several samples, such as P7 and P11 (Figure 1A,C). Detailed AGG interruption maps for additional samples are provided in Supplementary Figure S1.

These results demonstrate significant heterogeneity in the number and positional distribution of AGG interruptions among the samples. Such fine-scale characteristics were clearly resolved with allele-level resolution through the tLRS-*FMR1*. It is important to emphasize that the Coriell database lacks such interruption information entirely, and conventional TP-PCR methods are unable to accurately detect these important structural variations due to technical limitations. By overcoming the constraints of previous analytical approaches, tLRS-*FMR1* has, for the first time, provided a complete AGG interruption map for the national reference materials.

### 3.4. Evaluation of the Analytical Robustness and Limit of Detection (LOD) of the tLRS-FMR1

The robustness of the tLRS-*FMR1* for characterizing national reference materials was evaluated using intra-assay and inter-assay replicate testing. The intra-assay repeatability was assessed by testing all samples in triplicate runs on a Sequel II CNDx system, while the inter-assay reproducibility was evaluated by performing replicate sequencing on different instruments (Supplementary Table S3). Complete concordance in CGG repeat numbers was observed for normal and intermediate alleles in both analyses. In contrast, premutation alleles exhibited minor variations, and full mutation alleles showed greater fluctuations. This increased variability is attributable to amplification background noise, which escalates with longer CGG repeat tracts. Despite these fluctuations—which remained within an acceptable range—100% concordance was achieved across all replicates, demonstrating excellent technical robustness of this method.

LOD was determined using a series of two-fold dilutions prepared from three positive reference samples, resulting in concentrations of 10 ng/μL, 5 ng/μL, and 3 ng/μL. Each dilution level was tested in triplicate (Supplementary Table S4). The LOD was established at 3 ng/μL, a value well below the threshold of 10 ng/μL recommended by the National Institutes for Food and Drug Control (NIFDC) of China. This sensitive detection capability confirms the robustness of the tLRS-*FMR1* in achieving accurate genotyping even at low DNA concentrations.

## 4. Discussion

In this study, we developed and applied tLRS-*FMR1* to comprehensively characterize a national reference material panel for FXS. The results demonstrate that the tLRS-*FMR1* showed clear advantages over conventional techniques in the precise quantification of CGG repeats and resolution of AGG interruption patterns, while also exhibiting excellent robustness and a low limit of detection.

Compared with conventional methods, tLRS-*FMR1* showed clear advantages in the characterization of complex *FMR1* repeat expansions. TP-PCR is widely used for FXS testing but has limited capacity for accurately sizing long CGG repeat tracts and resolving AGG interruptions [27], whereas Southern blotting can identify full mutations and methylation status, but it is time-consuming, requires relatively large amounts of DNA, provides limited precision for CGG repeat sizing, and cannot define AGG interruption patterns [13,28]. In contrast, tLRS-*FMR1* directly interrogates the amplified *FMR1* repeat region using long-read sequencing, allowing simultaneous CGG repeat sizing and AGG interruption mapping. Compared with long-read genome sequencing, this targeted design concentrates sequencing depth on the *FMR1* locus, reducing unnecessary genome-wide sequencing and improving cost-effectiveness for reference material characterization. Beyond FXS, long-read sequencing has shown advantages in other genetically complex disorders, including congenital adrenal hyperplasia and thalassemia [29,30], and has been applied to the characterization of national genomic DNA reference materials for thalassemia variants [31]. Together, these findings support the broader potential of targeted long-read sequencing for complex variant analysis and genetic reference material development.

From a methodological perspective, this study validated the robust analytical performance of tLRS-*FMR1* for reference material value assignment. Intra- and inter-assay reproducibility experiments confirmed the high robustness of this method. Although longer CGG repeats introduce certain amplification background noise, leading to acceptable minor variations in the quantification of full mutation alleles, 100% genotyping concordance was maintained. Concurrently, the assay demonstrated high sensitivity, with an LOD of 3 ng/μL, which is significantly lower than the threshold recommended by the NIFDC. This indicates that reliable results can be obtained even from clinical samples with limited DNA input. Such high robustness and sensitivity are essential prerequisites for employing tLRS-*FMR1* as a value-assignment method for new-generation reference materials [32].

Furthermore, the new-generation national FXS reference material system established in this study has important implications for the standardization of FXS molecular diagnostics. Existing reference materials provide limited molecular annotations, particularly with respect to AGG interruption patterns, despite the established relevance of AGG interruptions to repeat stability and maternal transmission risk [10,33]. By integrating precise CGG repeat sizing with allele-level AGG interruption profiles, the reference material system characterized here provides a more comprehensive framework for analytical validation of emerging FXS testing technologies. In particular, it enables the evaluation and calibration of AGG interruption resolution, an analytical dimension that cannot be adequately assessed using existing reference materials [34]. Therefore, this system may serve as a traceable benchmark for clinical laboratories to validate PCR-, NGS-, and LRS-based FXS testing workflows, thereby supporting assay standardization, inter-laboratory comparability, and harmonization of results across technological platforms.

This study has certain limitations. Although we evaluated 31 candidate samples and included two authentic repeat-size mosaic samples in the final reference material panel, the overall sample size remains limited, especially for rare *FMR1* variant types [35]. Suitable samples with *FMR1* deletions or point variants were not available for inclusion in the current study. In addition, because tLRS-*FMR1* is based on PCR amplification, it is designed for CGG repeat sizing and AGG interruption analysis rather than methylation assessment. Therefore, methylation-specific PCR or Southern blot analysis remains necessary when methylation status is clinically required [36]. Future work should further expand the panel by incorporating clinically confirmed *FMR1* deletion, point-variant, and methylation-defined samples and further optimize the assay to improve the comprehensiveness of *FMR1* characterization.

## 5. Conclusions

This study developed and applied tLRS-*FMR1*, successfully constructing a new-generation national FXS reference material system with more comprehensive information and more precise characterization. This work not only highlights the considerable advantages of the tLRS-*FMR1* in the molecular diagnosis of complex repeat expansion disorders but also establishes a solid foundation for the precise molecular diagnosis and standardized quality control of FXS. Future expansion of the panel to include rare *FMR1* variant types and methylation-defined samples will further improve its clinical and standardization value.

## Figures and Tables

**Table 1:** Comparison of *FMR1* genotypes obtained from the Coriell Cell Repository, TP-PCR and the tLRS-*FMR1*.

Sample	Gender	Type	Coriell	TP-PCR	tLRS-*FMR1*	
			CGG	CGG	CGG	AGG
P1	M	I	46	45	46	9A36 *
P2	M	I	53	53	54	54
P3	F	N/PM	30/80	30/81	30/81	10A9A9/10A70
P4	F	N/PM	29/93–110	29/115	29/115	9A9A9/10A104
P5	F	N/PM	29/56	29/57	29/57	9A9A9/10A46
P6	M	PM	100–104	98	99	9A89
P7	F	N/FM	28–29/>200	29/>200	29/309	9A9A9/10A298
P8	M	FM	>200	>200	311	10A300
P9	M	FM	931–940	>200	900	900
P10	M	FM	501–550	>200	508	508
P11	M	PM/FM	100–104/>200	98/>200	98/311	9A88/10A300
P12	M	N/FM	29/501–550	29/>200	29/505	9A9A9/505
P13	F	N/PM/FM	23/95–140	23/134/>200	23/134/209	13A9/10A123/10A198
P14	M	FM	>200	>200	336/664	9A326/9A654
N1	F	N/N	NA	29/30	29/30	9A9A9/10A18/10A19
N2	M	N	NA	29	29	9A9A9
N3	F	N/N	NA	30/31	30/31	10A9A9/10A9A10
N4	F	N/N	29/41	29/41	29/41	9A9A9/10A9A20
N5	M	N	NA	30	30	9A20
N6	M	N	41	41	41	9A9A21
N7	NA	NA	NA	ND	ND	ND
N8	NA	NA	NA	ND	ND	ND

F: female; M: male; N: normal allele; I: intermediate allele; PM: premutation; FM: full mutation; NA: not applicable; ND: not detected; *: 9A36 was the abbreviation for (CGG)_9_AGG(CGG)_36_.

**Table 2:** Summary of AGG interruption patterns detected by the tLRS-*FMR1.*

Sample	CGG Repeat Number	AGG Interruptions Number	CGG-AGG Pattern	Read Count
P1	46	1 ^a^	9A36 ^b^	5082
P2	54	0	54	7878
P3	30	2	10A9A9	2234
P3	81	1	10A70	3970
P4	29	2	9A9A9	2456
P4	115	1	10A104	2195
P5	29	2	9A9A9	3022
P5	57	1	10A46	4160
P6	99	1	9A89	2472
P7	29	2	9A9A9	3108
P7	309	1	10A298	2348
P8	311	1	10A300	2290
P9	900	0	900	414
P10	508	0	508	718
P11	98	1	9A88	1533
P11	311	1	10A300	1475
P12	29	2	9A9A9	1480
P12	505	0	505	373
P13	23	1	13A9	30,815
P13	134	1	10A123	2198
P13	209	1	10A198	2320
P14	336	1	9A326	384
P14	664	1	9A654	49
N1	29	2	9A9A9	2444
N1	29	1	10A18	956
N1	30	1	10A19	2579
N2	29	2	9A9A9	1600
N3	30	2	10A9A9	4440
N3	31	2	10A9A10	3326
N4	29	2	9A9A9	4776
N4	41	2	10A9A20	3704
N5	30	1	9A20	7318
N6	41	2	9A9A21	2647
N7	ND	ND	ND	ND
N8	ND	ND	ND	ND

NA: not applicable; ND: not detected; ^a^: represents the number of AGG motifs within the *FMR1* CGG repeat tract. A value of 0 indicates no AGG interruption, while 1 and 2 indicate one and two AGG interruptions, respectively. ^b^: 9A36 was the abbreviation for (CGG)_9_AGG(CGG)_36_.

## Data Availability

The data generated or analyzed in this study are available in the published article and its supplementary files. The raw sequence data reported in this paper have been deposited in the Genome Sequence Archive (Genomics, Proteomics & Bioinformatics 2025) in the National Genomics Data Center (Nucleic Acids Res 2025), China National Center for Bioinformation/Beijing Institute of Genomics, Chinese Academy of Sciences (GSA-Human: HRA015938) that are publicly accessible at https://ngdc.cncb.ac.cn/gsa-human (accessed on 1 May 2026).

## Supplementary Materials

The following supporting information can be downloaded at https://www.mdpi.com/article/10.3390/genes17060656/s1, Figure S1: Analysis of the remaining FXS reference samples by the tLRS-*FMR1* and TP-PCR. Nucleotides: blue (CGG), orange (AGG), gray (other), the arrows highlight the alleles with different CGG repeats. Table S1. Candidate National Reference Materials for Fragile X Syndrome. Table S2. *FMR1* genotyping results from Coriell records, TP-PCR, and tLRS-*FMR1* for samples not included in the final reference material panel. Table S3. Intra- and inter-assay reproducibility of the tLRS-*FMR1*. Table S4. Limit of detection assessment of the tLRS-*FMR1*.

## Abbreviations

The following abbreviations are used in this manuscript:

ASD	Autism spectrum disorder
CCS	Circular consensus sequencing
FXS	Fragile X syndrome
FM	Full mutation
LOD	Limit of detection
LR-PCR	Long-range polymerase chain reaction
LRS	Long-read sequencing
PM	Premutation
tLRS-*FMR1*	Targeted long-read sequencing assay for comprehensive *FMR1* characterization

## References

[1] Mila M., Alvarez-Mora M.I., Madrigal I., Rodriguez-Revenga L. (2018). Fragile X Syndrome: An Overview and Update of the FMR1 Gene. Clin. Genet..

[2] Bagni C., Tassone F., Neri G., Hagerman R. (2012). Fragile X Syndrome: Causes, Diagnosis, Mechanisms, and Therapeutics. J. Clin. Investig..

[3] Liu X.S., Wu H., Krzisch M., Wu X., Graef J., Muffat J., Hnisz D., Li C.H., Yuan B., Xu C. (2018). Rescue of Fragile X Syndrome Neurons by DNA Methylation Editing of the FMR1 Gene. Cell.

[4] Salcedo-Arellano M.J., Dufour B., McLennan Y., Martinez-Cerdeno V., Hagerman R. (2020). Fragile X Syndrome and Associated Disorders: Clinical Aspects and Pathology. Neurobiol. Dis..

[5] Rousseau F., Rouillard P., Morel M.L., Khandjian E.W., Morgan K. (1995). Prevalence of Carriers of Premutation-Size Alleles of the FMRI Gene-and Implications for the Population Genetics of the Fragile X Syndrome. Am. J. Hum. Genet..

[6] Cabal-Herrera A.M., Tassanakijpanich N., Salcedo-Arellano M.J., Hagerman R.J. (2020). Fragile X-Associated Tremor/Ataxia Syndrome (FXTAS): Pathophysiology and Clinical Implications. Int. J. Mol. Sci..

[7] Fink D.A., Nelson L.M., Pyeritz R., Johnson J., Sherman S.L., Cohen Y., Elizur S.E. (2018). Fragile X Associated Primary Ovarian Insufficiency (FXPOI): Case Report and Literature Review. Front. Genet..

[8] Wheeler A.C., Bailey D.B., Berry-Kravis E., Greenberg J., Losh M., Mailick M., Milà M., Olichney J.M., Rodriguez-Revenga L., Sherman S. (2014). Associated Features in Females with an FMR1 Premutation. J. Neurodev. Disord..

[9] Nolin S.L., Brown W.T., Glicksman A., Houck G.E., Gargano A.D., Sullivan A., Biancalana V., Bröndum-Nielsen K., Hjalgrim H., Holinski-Feder E. (2003). Expansion of the Fragile X CGG Repeat in Females with Premutation or Intermediate Alleles. Am. J. Hum. Genet..

[10] Nolin S.L., Glicksman A., Ersalesi N., Dobkin C., Brown W.T., Cao R., Blatt E., Sah S., Latham G.J., Hadd A.G. (2015). Fragile X Full Mutation Expansions Are Inhibited by One or More AGG Interruptions in Premutation Carriers. Genet. Med..

[11] Yrigollen C.M., Durbin-Johnson B., Gane L., Nelson D.L., Hagerman R., Hagerman P.J., Tassone F. (2012). AGG Interruptions within the Maternal FMR1 Gene Reduce the Risk of Offspring with Fragile X Syndrome. Genet. Med..

[12] McConkie-Rosell A., Finucane B., Cronister A., Abrams L., Bennett R.L., Pettersen B.J. (2005). Genetic Counseling for Fragile X Syndrome: Updated Recommendations of the National Society of Genetic Counselors. J. Genet. Couns..

[13] Ciobanu C.G., Nucă I., Popescu R., Antoci L.M., Caba L., Ivanov A.V., Cojocaru K.A., Rusu C., Mihai C.T., Pânzaru M.C. (2023). Narrative Review: Update on the Molecular Diagnosis of Fragile X Syndrome. Int. J. Mol. Sci..

[14] Hayward B.E., Usdin K. (2017). Improved Assays for AGG Interruptions in Fragile X Premutation Carriers. J. Mol. Diagn..

[15] Coffee B., Ikeda M., Budimirovic D.B., Hjelm L.N., Kaufmann W.E., Warren S.T. (2008). Mosaic FMR1 Deletion Causes Fragile X Syndrome and Can Lead to Molecular Misdiagnosis: A Case Report and Review of the Literature. Am. J. Med. Genet. A.

[16] Rzónca S.O., Gos M., Szopa D., Sielska-Rotblum D., Landowska A., Szpecht-Potocka A., Milewski M., Czekajska J., Abramowicz A., Obersztyn E. (2016). Towards a Better Molecular Diagnosis of FMR1-Related Disorders—A Multiyear Experience from a Reference Lab. Genes.

[17] Tassone F. (2015). Advanced Technologies for the Molecular Diagnosis of Fragile X Syndrome. Expert Rev. Mol. Diagn..

[18] Biancalana V., Glaeser D., McQuaid S., Steinbach P. (2015). EMQN Best Practice Guidelines for the Molecular Genetic Testing and Reporting of Fragile X Syndrome and Other Fragile X-Associated Disorders. Eur. J. Hum. Genet..

[19] Dolzhenko E., van Vugt J.J.F.A., Shaw R.J., Bekritsky M.A., Van Blitterswijk M., Narzisi G., Ajay S.S., Rajan V., Lajoie B.R., Johnson N.H. (2017). Detection of Long Repeat Expansions from PCR-Free Whole-Genome Sequence Data. Genome Res..

[20] Stevanovski I., Chintalaphani S.R., Gamaarachchi H., Ferguson J.M., Pineda S.S., Scriba C.K., Tchan M., Fung V., Ng K., Cortese A. (2022). Comprehensive Genetic Diagnosis of Tandem Repeat Expansion Disorders with Programmable Targeted Nanopore Sequencing. Sci. Adv..

[21] Ardui S., Race V., Zablotskaya A., Hestand M.S., Van Esch H., Devriendt K., Matthijs G., Vermeesch J.R. (2017). Detecting AGG Interruptions in Male and Female FMR1 Premutation Carriers by Single-Molecule Sequencing. Hum. Mutat..

[22] Ek M., Kvarnung M., Ten Berk de Boer E., La Fleur L., Ljöstad L., Lyander A., Faergeman S.L., Drue S.O., Thonberg H., Nordgren A. (2026). Long-Read Genome Sequencing Enhances Diagnostics of Pediatric Neurological Disorders. Genome Med..

[23] Liang Q., Liu Y., Liu Y., Duan R., Meng W., Zhan J., Xia J., Mao A., Liang D., Wu L. (2022). Comprehensive Analysis of Fragile X Syndrome: Full Characterization of the FMR1 Locus by Long-Read Sequencing. Clin. Chem..

[24] Hou F., Mao A., Shan S., Li Y., Meng W., Zhan J., Nie W., Jin H. (2023). Evaluating the Clinical Utility of a Long-Read Sequencing-Based Approach in Genetic Testing of Fragile-X Syndrome. Clin. Chim. Acta.

[25] Gao F., Huang W., You Y., Huang J., Zhao J., Xue J., Kang H., Zhu Y., Hu Z., Allen E.G. (2020). Development of Chinese Genetic Reference Panel for Fragile X Syndrome and Its Application to the Screen of 10,000 Chinese Pregnant Women and Women Planning Pregnancy. Mol. Genet. Genom. Med..

[26] Hayward B.E., Kumari D., Usdin K. (2017). Recent Advances in Assays for the Fragile X-Related Disorders. Hum. Genet..

[27] Loomis E.W., Eid J.S., Peluso P., Yin J., Hickey L., Rank D., McCalmon S., Hagerman R.J., Tassone F., Hagerman P.J. (2013). Sequencing the Unsequenceable: Expanded CGG-Repeat Alleles of the Fragile X Gene. Genome Res..

[28] Shou X.Y., Zhu Z.W., Jin H., Hu J.H., Yan T.Z., Zhong Q.Y., Li W.H., Mao J.H., Dong M.Y., Xu Q. (2025). Enhanced Accuracy and Sensitivity in Detecting FMR1 CGG Repeats: A Multicenter Evaluation of a Novel PCR-Capillary Electrophoresis Assay. World J. Pediatr..

[29] Liu Y., Chen M., Liu J., Mao A., Teng Y., Yan H., Zhu H., Li Z., Liang D., Wu L. (2022). Comprehensive Analysis of Congenital Adrenal Hyperplasia Using Long-Read Sequencing. Clin. Chem..

[30] Liang Q., He J., Li Q., Zhou Y., Liu Y., Li Y., Tang L., Huang S., Li R., Zeng F. (2023). Evaluating the Clinical Utility of a Long-Read Sequencing-Based Approach in Prenatal Diagnosis of Thalassemia. Clin. Chem..

[31] Wei X., Yang X., Han W., Zhang L., Ouyang G., Qu S., Yang F., Yang X. (2025). Applying the National Genomic DNA Reference Materials to Evaluate the Performance of Nanopore Sequencing in Identifying Thalassemia Variants. J. Clin. Lab. Anal..

[32] (2024). Reference Materials—Approaches for Characterization and Assessment of Homogeneity and Stability.

[33] Nolin S.L., Sah S., Glicksman A., Sherman S.L., Allen E., Berry-Kravis E., Tassone F., Yrigollen C., Cronister A., Jodah M. (2013). Fragile X AGG Analysis Provides New Risk Predictions for 45-69 Repeat Alleles. Am. J. Med. Genet. A.

[34] Hawkins M., Boyle J., Wright K.E., Elles R., Ramsden S.C., O’Grady A., Sweeney M., Barton D.E., Burgess T., Moore M. (2011). Preparation and Validation of the First WHO International Genetic Reference Panel for Fragile X Syndrome. Eur. J. Hum. Genet..

[35] Quartier A., Poquet H., Gilbert-Dussardier B., Rossi M., Casteleyn A.S., Portes V.D., Feger C., Nourisson E., Kuentz P., Redin C. (2017). Intragenic FMR1 Disease-Causing Variants: A Significant Mutational Mechanism Leading to Fragile-X Syndrome. Eur. J. Hum. Genet..

[36] Spector E., Behlmann A., Kronquist K., Rose N.C., Lyon E., Reddi H.V. (2021). Laboratory Testing for Fragile X, 2021 Revision: A Technical Standard of the American College of Medical Genetics and Genomics (ACMG). Genet. Med..

