# Supplementary material for: Establishment of a New-Generation National Reference Material System for Fragile X Syndrome Using Targeted Long-Read Sequencing
Source: Genes (Basel). 2026 Jun 2;17(6):656. doi: 10.3390/genes17060656 (PMC13299665; doi:10.3390/genes17060656)
Supplement: Supplementary file 1 [file genes-17-00656-s001.zip › genes-4342790-supplementary.pdf]

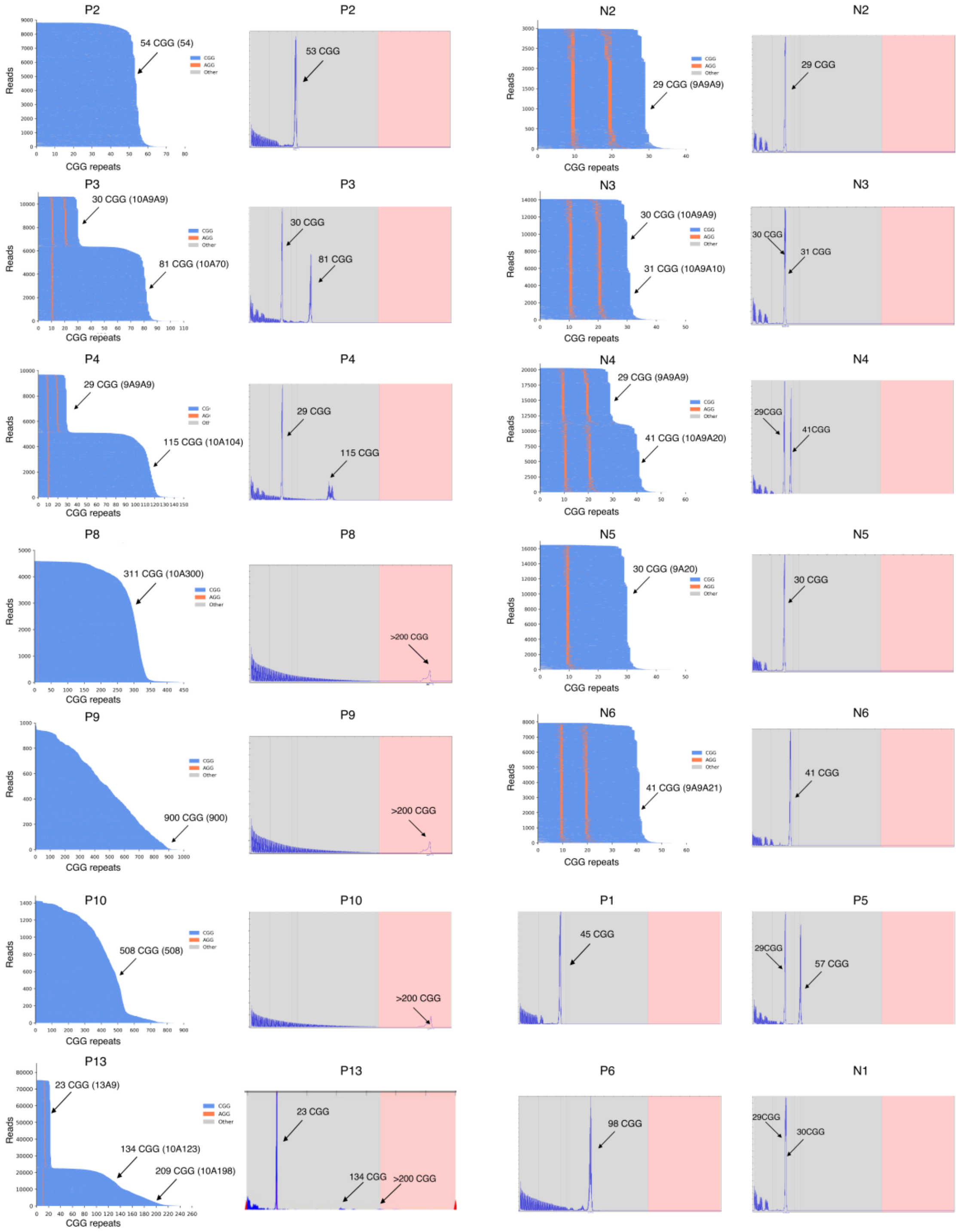

Supplementary Figure S1. Analysis of the remaining FXS reference samples by the tLRS-*FMR1* and TP-PCR. Nucleotides: blue (CGG), orange (AGG), gray (other), the arrows highlight the alleles with different CGG repeats.

Supplementary Table S1. Candidate National Reference Materials for Fragile X Syndrome.

No.	Sample	ID	Gender	Type	CGG repeats
1	P1	GM20232	Male	Intermediate	46
2	P2	GM20230	Male	Intermediate	53
3	P3	FXS002	Female	Normal/Premutation	30/80
4	P4	GM20241	Female	Normal/Premutation	29/93-110
5	P5	FXS031901	Female	Normal/Intermediate	29/56
6	P6	GM20237	Male	Premutation	100-104
7	P7	GM07537	Female	Normal/Full Mutation	28-29/>200
8	P8	GM06852	Male	Full Mutation	>200
9	P9	GM09237	Male	Full Mutation	931-940
10	P10	GM07862	Male	Full Mutation	501-550
11	P11	GM20237&GM06852	Male	Mosaic	100-104/>200
12	P12	GM24631&GM07862	Male	Mosaic	29/501-550
13	P13	GM06896	Female	Normal/Premutation	23/95-140
14	P14	GM04026	Male	Full Mutation	>200
15	N1	GM24695	Female	Normal	NA
16	N2	GM24631	Male	Normal	NA
17	N3	GM12878	Female	Normal	NA
18	N4	GM20243	Female	Normal	29/41
19	N5	GM24694	Male	Normal	NA
20	N6	GM20244	Male	Normal	41
21	N7	*Escherichia coli* DNA1	NA	NA	NA
22	N8	*Escherichia coli* DNA2	NA	NA	NA
23	S1	GM06897	Male	Full Mutation	>200
24	S2	GM20239	Female	Normal/Premutation	20/193
25	S3	GM03200	Male	Full Mutation	477
26	S4	GM04025	Male	Full Mutation	645
27	S5	GM06891	Male	Premutation	118
28	S6	GM06894	Female	Normal/Premutation	30/78
29	S7	GM06903	Female	Normal/Premutation	23/95
30	S8	GM06910	Female	Normal/Premutation	30/75-89
31	S9	GM09316	Male	Full Mutation	>200

NA: not applicable

Supplementary Table S2. *FMR1* genotyping results from Coriell records, TP-PCR, and tLRS-*FMR1* for samples not included in the final reference material panel.

Sample	ID	Type	Coriell	TP-PCR	tLRS-*FMR1*	
			CGG	CGG	CGG	AGG
S1	GM06897	FM	>200	>200	333	10A322*
S2	GM20239	N/PM	20/193	20/193	20/193	10A9/9A9A170
S3	GM03200	FM	477	>200	485	485
S4	GM04025	FM	645	>200	571	571
S5	GM06891	PM	118	125	120	10A109
S6	GM06894	N/PM	30/78	30/81	30/82	10A9A9/10A71
S7	GM06903	N/PM	23/95	24/93	24/93	14A9/10A82
S8	GM06910	FM	30/75-89	30/87	30/88	10A9A9/10A77
S9	GM09316	FM	>200	>200	310	9A300

N: normal allele; I: intermediate allele; PM: premutation; FM: full mutation; NA: not applicable; ND: not detected; *: 10A322 was the abbreviation for $(CGG)_{10}AGG(CGG)_{322}$.

Supplementary Table S3. Intra- and inter-assay reproducibility of the tLRS-*FMR1*.

Sample	Gender	Sequel II CNDx			PacBio Vega			PacBio Sequel IIe	
		R1	R2	R3	R1	R2	R3	R1	R2
P1	M	46	46	46	/	/	/	46	46
P2	M	54	54	54	/	/	/	54	54
P3	F	30/81	30/81	30/81	30/81	30/81	/	/	/
P4	F	29/115	29/115	29/115	/	/	/	29/115	29/116
P5	F	29/57	29/57	29/57	/	/	/	29/57	29/57
P6	M	99	100	99	/	/	/	100	100
P7	F	29/309	29/308	29/309	/	/	/	29/316	29/314
P8	M	311	312	311	/	/	/	311	310
P9	M	900	900	901	920	924	919	/	/
P10	M	508	504	505	497	500	499	485	490
P11	M	99/311	98/311	100/310	96/306	96/306	97/306	/	/
P12	M	29/505	29/507	29/513	29/491	29/490	29/495	/	/
P13	F	23/134 /208	23/13 /209	23/134 /210	/	/	/	/	/
P14	M	120	120	122	/	/	/	/	/
N1	F	29/30	29/30	29/30	/	/	/	29/30	29/30
N2	M	29	29	29	/	/	/	29	29
N3	F	30/31	30/31	30/31	/	/	/	30/31	30/31
N4	F	29/41	29/41	29/41	/	/	/	29/41	29/41
N5	M	30	30	30	/	/	/	30	30
N6	M	41	41	41	/	/	/	41	41
N7	NA	ND	ND	ND	/	/	/	/	/
N8	NA	ND	ND	ND	/	/	/	/	/

F: female; M: male; N: normal allele; I: intermediate allele; PM: premutation; FM: full mutation; NA: not applicable; ND: not detected; "/", not tested.

Supplementary Table S4. Limit of detection assessment of the tLRS-*FMR1*.

Sample	Concentration gradient	CGG repeat		
		R1	R2	R3
P2	10 ng/μL	53	53	53
	5 ng/μL	53	53	53
	3ng/μL	53	53	53
P7	10 ng/μL	29/304	29/302	29/304
	5 ng/μL	29/302	29/304	29/303
	3ng/μL	29/303	29/304	29/303
P12	10 ng/μL	29/484	29/493	29/486
	5 ng/μL	29/497	29/492	29/488
	3ng/μL	29/488	29/502	29/503
